# Impact of “Sick” and “Recovery” Roles on Brain Injury Rehabilitation Outcomes

**DOI:** 10.1155/2012/725078

**Published:** 2012-10-15

**Authors:** David A. Barclay

**Affiliations:** Department of Social Work, Gallaudet University, 800 Florida Avenue NE, Washington, DC 20008, USA

## Abstract

This study utilizes a multivariate, correlational, expost facto research design to examine Parsons' “sick role” as a dynamic, time-sensitive process of “sick role” and “recovery role” and the impact of this process on goal attainment (H1) and psychosocial distress (H2) of adult survivors of acquired brain injury. Measures used include the Brief Symptom Inventory-18, a Goal Attainment Scale, and an original instrument to measure sick role process. 60 survivors of ABI enrolled in community reentry rehabilitation participated. Stepwise regression analyses did not fully support the multivariate hypotheses. Two models emerged from the stepwise analyses. Goal attainment, gender, and postrehab responsibilities accounted for 40% of the shared variance of psychosocial distress. Anxiety and depression accounted for 22% of the shared variance of goal attainment with anxiety contributing to the majority of the explained variance. Bivariate analysis found sick role variables, anxiety, somatization, depression, gender, and goal attainment as significant. The study has implications for ABI rehabilitation in placing greater emphasis on sick role processes, anxiety, gender, and goal attainment in guiding program planning and future research with survivors of ABI.

## 1. Introduction

Based on nonmilitary hospital, emergency room, and death records, an estimated 1.7 million Americans suffer a traumatic brain injury (TBI) each year [1]. Additional US military cases of TBI total over 20,000 annually [2]. Add to that number the additional annual 700,000 stroke victims [3], and the prevalence of people suffering any type of acquired brain injury (ABI) totals over 2.5 million Americans annually. For purposes of perspective, each year 12,000 people suffer a traumatic spinal cord injury [4], 50,000 are diagnosed with AIDS, and 176,000 are diagnosed with breast cancer [5]. Following medical treatment for acquired brain injury (ABI), many persons continue treatment in outpatient community reentry rehabilitation programs. Upon discharge, however, many survivors of ABI fail to demonstrate optimal goal attainment [6] and suffer heightened psychosocial distress as demonstrated by increased depression and anxiety [7–11]. Although there is not a consensus in the literature related to the prevalence of depression and anxiety, the number of brain injury survivors who suffer from depression can be as high as 77% and those who suffer from anxiety can be as high as 66% of the brain injury survivors studied [7–11]. Two recent studies emphasize the amount and longevity of depression in brain injury survivors. Jorge et al. [12] found that over one-third of their sample survivors met the criteria for major depressive disorder and over two thirds met the criteria for an anxiety disorder. According to Konrad et al. [13], depression and anxiety continue to be an issue many years postinjury. Their study sample of 33 mild TBI survivors 6 years postinjury had significantly higher depression scores as measured by the Beck Depression Inventory than the general population.

Social and community integration can be severely hampered if individual rehabilitation goals are not met [14] and if the survivor continues to experience psychosocial distress, specifically anxiety and depression. Much of the current literature suggests that a brain injury survivor's quality of life suffers if he/she does not achieve optimal goal attainment at time of discharge and if he/she continues to experience anxiety and depressive symptoms at time of discharge. Often, social and emotional behavior impairments are considered by the survivor as more disabling than the physical residuals of the injury [15]. These psychosocial behavioral impairments can negatively impact neuropsychological functioning [16] and can impede psychosocial adjustment following rehabilitation [17, 18]. Increases in depression and anxiety are a common residual problem for the brain injury survivor [19, 20] and have been shown to impact cognitive recovery, return to work/school and the family system [21–26].

In understanding human behavior, both the structure of a role and the process of the assumption of a role are important [27]. In examining possible explanations for poor goal attainment and continued psychosocial distress after rehabilitation, Talcott Parsons' sick role theory can be applied as a framework for understanding illness behavior and outcome pathways. Sick role is conceptually defined as one's acceptance of certain rights and responsibilities associated with the role of being sick. Parsons [26] emphasized the impact that society has on the structure of roles. Societal and self-generated expectations and legitimations of the role, in particular, are important to the understanding and to the role-taking process. Thus part of the process of experimentation with and acceptance of new roles is whether the self and/or society accepts that role and in what condition [26, 27]. Environmental elements and both self and society can reinforce the structure and process of a role by either the nurturing or rejection of a role. Thus, the physical rehabilitation environment can directly control role assumption through availability of resources, and the ABI survivor's social environment can control role assumption through direct commands or interpersonal behaviors [27].

Parson's original blueprint of the sick role [26, 28] was restricted to societal norms that influenced a person's role during temporary, acute physical illness. The four major tenets of his sick role concept are (1) the sick person is exempt from social responsibility, (2) the sick person is exempt from self-blame for being sick, (3) the sick person should want to get well, and (4) the sick person should seek medical advice and cooperate with medical experts [26]. Parsons combined these rights and responsibilities to form a one-dimensional set of societal expectations called the “sick role.” Sociologically, his main concern was how the sick role prevented individuals from performing their tasks in society and whether certain parts of society had more or less of a tendency to assume and/or condone assumption of the sick role. 

Early research on assumption of the sick role focused on societal perceptions of sick people; in particular people who had an acute illness [29]. The research focused on whether they, from the perception of others, had a legitimate right to assume the sick role. However, during the decades that followed, Parsons' model has been extended beyond perceptions of those who had temporary, acute illness to include patterns in the actual ill person's assumption and relinquishment of the sick role. In addition, studies were expanded to include persons with chronic illness and psychiatric illness. Several studies focused on variables impacting a person's willingness to adopt the sick role, rather than on the expectations that society holds towards those people [30–33].

This current study investigates the sick role as a process of assumption and relinquishment of rights and responsibilities. If the sick role is framed as a process of rights and responsibilities, then a person may take one of three common paths with regards to the sick role: (1) he or she may accept the sick role rights when appropriate and then assume the necessary responsibilities of therapy and recovery, and then relinquish the sick role, or (2) he or she may reject the sick role rights and responsibilities in denial of his/her illness and thus be in denial of any limitations or requirements for therapy, or (3) he or she may overidentify with the rights of the sick role, exemption from responsibility, and dependence on others, without desiring to get well and without seeking and cooperating with therapies.

Currently, no studies directly apply sick role concepts to the ABI survivor rehabilitation process. However, several articles apply the sick role to other populations (e.g., cardiac patients) who follow a similar path of ABI survivors in terms of the seriousness and suddenness of their change in abilities. In the cardiac population, age and gender were significant predictors of sick role tendencies [30–33]. In particular, men tended to relinquish the sick role more readily than women and younger people tended to relinquish the sick role more readily than older people [32, 33]. Because the literature indicates the variables of age and gender affect assumption of the sick role, they are taken into account as possible external sources of variance in the present research project.

## 2. Methods

### 2.1. Design

This study utilized a multivariate, correlational, expost facto research design. Control variables were age, gender, and level of functioning. The study sought to answer the following question. What is the relationship between the independent variables of survivor sick role process and the dependent variables of goal attainment and psychosocial distress in adults with acquired brain injuries involved in the rehabilitation process? The following two hypotheses were developed to help answer this question.

**Hypothesis 1**

Controlling for age and gender, those survivors of ABI with higher levels of acceptance of their sick role rights at the beginning of rehabilitation and higher levels of acceptance of their sick role responsibilities upon discharge will have lower levels of psychosocial distress.

**Hypothesis 2**

Controlling for age and gender, those survivors of ABI with higher levels of acceptance of their sick role rights at the beginning of rehabilitation and higher levels of acceptance of their sick role responsibilities upon discharge will have higher levels of goal attainment.

### 2.2. Participants

Participants were 60 adult survivors of acquired brain injury (ABI) who attended outpatient community reentry rehabilitation at one of two programs located in the Washington, DC, metropolitan area in the USA. For the purposes of this study, an ABI survivor was defined as someone who had experienced any insult to the brain which resulted in impairment of cognitive abilities and/or physical functioning [34]. 

The study utilized a convenience sample of adult (18 years of age or over) survivors of ABI. Candidates were referred and screened by their primary therapists in their rehabilitation program. Candidates were not eligible if there was evidence of severe memory deficits or an unmanaged mood or substance abuse disorder. To further control for possible memory impairments impacting the validity of this study, after referral to the study, participants were asked to self-report on their ability to recall events and feelings that they experienced at the beginning of their rehabilitation in retrospect. Participation was voluntary, although a $10 compensation was given to all participants. A written informed consent form was reviewed with all candidates. Prior to participating, all participants signed the informed consent forms which were preapproved by two separate Institutional Review Boards (The Catholic University of America and Gallaudet University, Washington, DC).

### 2.3. Data Collection

This research was part of a larger research study conducted as part of the dissertation process for completion of doctoral studies. Questionnaires were administered to the participants over a 15-month period. Individual survivor goals were established for each survivor by the rehabilitation agency based on agency assessment. The 54-item study instrument for this research study consisted of demographic questions, two previously published instruments, and one original instrument. The demographic questionnaire contained 13 closed-ended questions about the control variables of age and gender and included additional questions about marital status, injury, income, education, and spiritual support. 

### 2.4. Barclay Sick Role Process Inventory (BSRPI)

The independent variable of level of sick role is conceptualized as the survivor's process of acceptance of sick role rights and responsibilities from the beginning to the end of the period of rehabilitation. No published measures specifically addressed the sick role rights and responsibilities in adult ABI survivors, so the researcher developed one for the study. The study uses a new scale, named the Barclay Sick Role Process Inventory (BSRPI) (see Appendix (A)) to measure these levels of acceptance of sick role rights and responsibilities over time. This new sick role process scale was adapted from Myers and Grasmick's [35] instrument measuring the static concept of sick role in pregnancy.

The BSRPI is a 24-item instrument. The completed scale has 12 items phrased in the past, “When I first started in this rehabilitation program …,” and 12 items phrased in the present, “Now …,” in order to identify the rights and responsibilities acceptance process. Item responses are rated on a 4-point scale ranging from (1) strongly disagree to (4) strongly agree. Data from this inventory can be analyzed to reflect individual and total rights and responsibilities both before and after rehabilitation and to reflect the change in acceptance of rights and responsibilities over time. 

Content validity was determined using a review of the literature and a panel of experts in the field of brain injury and mental health. In addition, internal consistency of items on the BSRPI was analyzed using Cronbach's alpha. The reliability alpha in the sample (*n* = 60) for the entire instrument was .70. However, the instrument actually measures four distinct concepts. The reliability alpha in the sample (*n* = 60) for each concept is as follows: .69 for individual rights at time of intake; .88 for individual responsibilities at time of intake; .58 for individual rights at time of discharge; .79 for individual responsibilities at time of discharge.

One concern of the use of collecting retrospective data from ABI survivors is the fact that memory problems are a common issue with this population. As a control for this validity concern, participants were accepted only upon referral by their primary therapists in the rehabilitation agencies. The therapists were instructed not to refer anyone with evidence of severe memory deficits or an unmanaged mood or substance abuse disorder. As an additional control, participants were asked the following two screening questions. (1) Do you remember how you were feeling when you were first admitted to this rehabilitation program? (2) Do you remember how you felt when you first met your rehabilitation therapists? They were also asked to identify and clarify a few of their identified feelings to further validate their answers. If they answered “no” to either question, they were screened out of the participant pool.

### 2.5. Rating Scale for Functional Independence (RSFI)

Level of functioning was measured by the Rating Scale for Functional Independence (RSFI) to control for the impact of level of functioning at time of intake on the participants' goal attainment and psychosocial distress. Level of functioning is conceptually defined as a subject's overall physical, cognitive, psychosocial, and behavioral ability to function. Both agencies where participants were recruited from utilized the RSFI. The RSFI is a way for the therapist to rate the functional independence of their client on a 7-point scale as follows: 1 = total assistance, 2 = maximal assistance, 3 = moderate assistance, 4 = minimal assistance, 5 = supervision, 6 = modified independence, and 7 = complete independence. There is a rubric with details on the measurable differences between each level of independence. The RSFI has not been studied related to reliability or validity. The participants' level of functioning at intake and discharge was assessed by the primary therapists with responses ranging from 1 to 7 with the ability to assess levels at .5 increments. 

### 2.6. Brief Symptom Inventory-18 (BSI)

Psychosocial distress, specifically depression and/or anxiety, was measured by the Brief Symptom Inventory-18 (BSI). This tool measures depression and anxiety and has alpha coefficients of greater than .90 [36]. The BSI is an 18-item instrument designed to measure psychological distress both during and at the end of treatment based on the three dimensions of depression, anxiety, and somatization. Individual scores are provided for each dimension along with a Global Severity Index (GSI) score which represents the overall level of psychological distress.

### 2.7. Goal Attainment Scale (GAS)

Goal attainment is conceptually defined as the level of success in meeting individualized goals upon discharge that were established at the beginning of the ABI survivor's rehabilitation program. Operationally, this was measured by a Goal Attainment Scale [37] generated by the primary therapists (see Appendix (B)) for the participants' cognitive, psychosocial, occupational, and physical rehabilitation. Overall goal attainment was recorded on a 5-point scale from most unfavorable outcome (1) to most favorable outcome (5). The primary therapists at the agency responsible for establishing and monitoring various goals were asked to give a numerical rating of each subject's goal attainment in each therapy discipline. These scores were then averaged across the number of individual rehabilitation goal areas for each participant.

## 3. Results

### 3.1. Sociodemographic Findings

Forty-seven of the 60 participants, or 78% of the sample, self-identified as males and 13, or 22% of the sample, self-identified as females. Participants ranged in age from 18 to 83 years of age, with the mean age being 43.6 years old. The reported racial/ethnic composition was as follows: 61% self-identified as Caucasian/White (non-Hispanic), 31.7% self-identified as African American/Black, 5% self-identified as Hispanic, and 1.7% self-identified as Asian/Pacific Islander. The participants had sustained the following type of injuries: 35% were due to stroke, 43% were due to acceleration/deceleration injury, and 22% were due to an “other” category which included alcoholic seizure, tumor, surgery, and encephalitis.  Table 1 illustrates the sociodemographic data described above.

### 3.2. Descriptive Statistics for the Study Instruments

Table 1 shows the descriptive statistics for the Barclay Sick Role Process Inventory (BSRPI), Brief Symptom Inventory-18 (BSI-18), and Goal Attainment Scale (GAS). Norms are not available for the BSRPI scales. However, the BSRPI responsibility at discharge actual range is smaller than the potential range and the mean score is fairly high. This signifies that the study sample scored at the high end of the scale which would be expected in the sick role process. This is conceptually significant because, according to sick role theory, the majority of the sample tended to assume high levels of sick role responsibilities at discharge. The BSRPI reliability is good with an alpha of .69 for measuring rights and an alpha of .79 for measuring responsibilities. 

Participants had a BSI-18 mean raw score of 7.52 which is only slightly higher than both the community and oncology raw score norms published by the author of the BSI-18 [38]. This signifies that the study sample reported slightly higher levels of psychosocial distress than samples in the general community and the cancer community. This could be explained by the added potential for psychosocial distress in the ABI community. The BSI-18 reliability for the study sample is very good with an alpha of .84.

The participants had a mean score of 3.04 on the GAS. Norms are not available for the GAS. However, a mean score of 3.04 signifies that the primary therapists reported that the study sample generally achieved their predicted level of goal attainment at an expected level. As shown in Table 2, the potential range (1–5) and the actual range (1.25–4.6) are very similar, signifying the therapists did indeed assign goal attainment ratings all along the achievement continuum. Among all of the variable measures, the GAS had the lowest reliability coefficient (.67). 

### 3.3. Bivariate Analysis

Pearson's product-moment correlation was used to investigate bivariate relationships among variables. Table 2 depicts the bivariate correlations among goal attainment (Goal), psychosocial distress total (PsyTot), somatization (Som), depression (Dep), anxiety (Anx), assumption of sick role rights before (PreRig) and after (PostRig) rehabilitation, assumption of sick role responsibilities before (PreRes) and after (PostRes) rehabilitation, level of functioning (LOF), time after injury in months (Time), age (Age), and gender (Gen). As can be seen in Table 2, goal attainment was significantly negatively correlated with total psychosocial distress (*r* = −.518, *P* < .01) and also with all three subscales of somatization (*r* = −.277, *P* < .01), depression (*r* = −.386, *P* < .01), and anxiety (*r* = −.439, *P* < .01). However, the analysis suggests that there is no significant relationship between goal attainment and any other variable, including sick role process variables.

Total psychosocial distress was significantly positively correlated with postrehab sick role rights (*r* = .310, *P* < .05) and gender (*r* = .428, *P* < .01). Anxiety was significantly positively correlated with goal attainment (*r* = −.439, *P* < .01) and gender (*r* = .570, *P* < .01) and postrehab sick role rights (*r* = .443, *P* < .01) and significantly negatively correlated with postrehab sick role responsibilities (*r* = −.388, *P* < .01). Somatization was significantly positively correlated with both pre- and postrehab sick role rights (*r* = .298, *P* < .05); (*r* = .315, *P* < .05) and gender (*r* = .430, *P* < .01). Depression was only significantly correlated with prerehab sick role responsibilities (*r* = −.338, *P* < .05). The assumption of sick role responsibilities at time of discharge is negatively correlated with both age (*r* = −.37, *P* < .01) and level of functioning (*r* = −.32, *P* < .05). 

### 3.4. Multivariate Analysis

Controlling for age and gender, higher levels of acceptance of sick role rights at the beginning of rehabilitation and higher levels of acceptance of sick role responsibilities upon discharge were hypothesized to predict lower levels of psychosocial distress in H1 and higher levels of goal attainment in H2. In order to examine the relative predictive contribution of the independent variables on the dependent variables, two stepwise regression analyses were conducted based on the hypotheses and significant relationships found in the bivariate analyses. Related to H1, the first stepwise regression analysis examined the contribution of the hypothesized sick role process variables, the control variables of age and gender, and the significant variable of goal attainment as predictor variables for psychosocial distress. Tests for multicollinearity indicated that a very low level of multicollinearity was present (VIF numbers for all variables were less than 1.5). As can be seen in Table 3, the model summary included goal attainment, gender, and postrehab responsibilities accounting for 40% of the shared variance of psychosocial distress with goal attainment contributing the majority of the explained variance. Pre-rehab sick role rights and responsibilities, postrehab rights, and age were excluded from the model. The observed power for this multiple regression model, given an alpha of.05, *n* of 60, and 6 predictor variables, is .99. H1 was not fully supported by the analysis, with only postrehab responsibilities being a significant predictor variable for psychosocial distress in this model. 

The second stepwise regression analysis examined the contribution of the hypothesized sick role process variables, the control variables of age and gender, and the significant variables of depression, anxiety, and somatization. Tests for multicollinearity indicated that a very low level of multicollinearity was present (VIF numbers for all variables were less than 1.2). As can be seen in Table 4, the model summary included anxiety and depression accounting for 22% of the shared variance of goal attainment with anxiety contributing to the majority of the explained variance. All sick role variables, gender, and age were excluded from the model. The observed power for this multiple regression model, given an alpha of .05, *n* of 60, and 9 predictor variables, is .80. H2 was not supported by the analysis; no variable of sick role process was found to be a significant predictor of goal attainment in this model.

## 4. Discussion

This study pioneers the application of sick role theory and expansion of role theory on brain injury rehabilitation. Theoretically, this study expanded upon Parsons' static sick role and applied it as a more dynamic process of assumption and relinquishment of sick role rights and responsibilities. An instrument was developed to help measure this sick role process. However, using stepwise regression analysis, the data did not fully support either research hypothesis related to sick role process. Consequently, controlling for age and gender, survivors of ABI with higher levels of acceptance of their sick role rights at the beginning of rehabilitation and higher levels of acceptance of their sick role responsibilities upon discharge did not significantly demonstrate lower levels of psychosocial distress nor higher levels of goal attainment. Although the data did not support the full hypotheses related to sick role process, several important significant relationships were uncovered related to sick role variables and other variables that can be used to better understand and improve rehabilitation with survivors of ABI. The most interesting relationships uncovered have to do with those between goal attainment and psychosocial distress, especially when examined as being potentially both independent and dependent variables in the rehabilitation process. Bivariate analysis showed a moderate significant negative relationship between psychosocial distress and goal attainment, but what is not clear is if the survivor was not achieving their goals due to their psychosocial distress or if the lack of goal achievement was increasing their psychosocial distress, or both. If psychosocial distress is identified as the dependent variable, goal attainment explained the majority of the variance in psychosocial distress, followed by gender and then assumption of postrehab rights. The literature supports the findings of goal attainment on psychosocial distress [19, 21] and emphasizes the need for establishing achievable goals and monitoring psychological and emotional status of the survivors, especially for those who have not achieved their rehabilitation goals. 

 To more accurately understand how psychosocial distress factors into the rehabilitation process and outcomes, it can be broken down into its three measurable components of depression, anxiety, and somatization and then the various relationships examined. In the regression model in Table 4, anxiety explained the majority of variance in goal attainment, followed by depression. In the bivariate analysis, participants who reported higher levels of anxiety not only had lower levels of goal attainment, they also had higher levels of sick role rights both before and after rehab, lower levels of postrehab sick role responsibilities, and tended to be female. Related to sick role process, these correlations support a profile of more anxious survivors who stay in the dependent role of “sick person” and who are not moving into the role of “recovering person.” The majority of the literature reviewed focus on the role of depression in the rehabilitation outcomes, but the results of the present study point to anxiety as a more important factor. Depression had a significant negative correlation only with goal attainment and prerehab sick role responsibilities, suggesting a relationship between higher levels of depression and lower levels of sick role “recovery” responsibilities at the beginning of rehabilitation. 

Similar to anxiety, somatization was significantly positively correlated with both pre- and postrehab sick role rights and gender. Those survivors who reported high levels of somatic symptoms tended also to enter rehab and exit rehab with high levels of dependent “sick role” rights and be female. Although the somatization subscale is designed to collect data on somatic symptoms associated with psychological stress (i.e., sleep problems, nausea), the instrument does not differentiate nor expand on the causes of the somatic symptoms. Brain injury survivors often have somatic symptoms that are due to their physical injuries and not to their psychological issues. So the subscale results cannot be unilaterally categorized nor analyzed as being “psycho-somatic.” However, what is clear is that there is a relationship between a survivors somatic symptoms and their initial and continued acceptance of sick role rights. Related to ABI rehabilitation, health care providers should be focusing on the reduction of somatic symptoms and consider them in relation to survivors moving from dependence towards recovery. 

In the study, gender was positively correlated with assumption of sick role rights both before and after rehab, psychosocial distress, somatization, and anxiety, meaning that women tended to assume sick role rights and have higher levels of various psychosocial distress than men. The literature supports that women tend to assume the static sick role of both rights [32]; however, the literature does not support that female ABI survivors tend to have higher levels of psychosocial distress than male ABI survivors. This correlation would need to be addressed in further research due to the small number of women in the study sample (*n* = 13). However, if the correlation that women tend to have greater psychosocial distress, anxiety, or somatization at time of discharge is further supported, then that would increase support for consideration of gender during intake and for program planning and discharge.

In the study, age had a significant negative correlation with sick role responsibilities at time of discharge (*r* = −.37, *P* < .01) and a significant positive correlation with assumption of sick role rights at time of intake (*r* = .34, *P* < .01). The literature suggests that younger people tend to accept the static sick role at the beginning of rehabilitation and tend to relinquish the static sick role at the end of rehabilitation more than older people [32]. Examined as a process, the data for this study suggests that older people tended to assume more sick role rights than younger people but then did not relinquish those rights and move onto accepting the sick role responsibilities. These correlations support the need for program planning that encourages older adults' assumption of sick role responsibilities.

Future research in the area of community reentry rehabilitation for adults survivors of ABI is needed to improve their short-term rehabilitation outcomes and long-term community reentry outcomes. This research should be used in conjunction with other empirical and theoretical literature to ultimately improve the quality of life of adult survivors of traumatic brain injury. Future research can use the results of this study as a foundation to build upon the idea of the sick role as a process. Sick role process was defined in the current study by measuring only two of the four sick role variables, prerehab sick role rights, and postrehab sick role responsibilities. Future research can utilize the four sick role variables which all had significant bivariate relationships with other variables, especially anxiety, goal attainment, depression, age, and gender. Operationally, the concept of sick role rights at the beginning of the rehabilitation should be examined further in future research due to the moderate reliability of the instrument that measured that specific concept. In addition, the instrument was memory dependent and this should be a consideration in future research.

### 4.1. Limitations of the Study

The present study has some methodological limitations that weaken the ability to generalize findings beyond the scope of this particular sample group. Sampling technique, sample size, instrumentation, control of independent, and human error are all variables which limit this study's generalizability.

The technique of convenience sampling was utilized due to limitations of resources. Randomization was not used which is a main quantitative tool for control of extraneous variables. The small sample size (*n* = 60) also weakened the power of the analysis when multiple variables were analyzed within the sample. Related to instrumentation, although the BSRPI was developed with consultation from brain injury experts, validity would have been increased if the experts were also experts on the concept of sick role. Reliability would have been increased if the BSRPI had been pilot tested several times prior to its use. ABI survivors sometimes have limited insight into their own problems due to cognitive processing which is a threat to internal validity, although screening methods were used to attempt to control for this threat.

Control variables that were derived from the prior literature were utilized to reduce the possibility of external sources of variance. However, there are possibly other independent variables that were not considered that were not in prior literature. Lastly, this researcher was the only person who collected data, which increased interrater reliability. However, human error is always a possibility when collecting data and inputting and analyzing data.

## Figures and Tables

**Table 1:** Summary statistics of the study scales.

	Mean	SD	Alpha	Potential range	Actual range
BSRPI*—rights at intake	12.65	2.82	.69	5–20	7–20
BSRPI—responsibility at discharge	21.80	2.54	.79	6–24	15–24
Brief Symptom Inventory: SI-18	7.52	7.08	.84	0–72	0–32
Goal Attainment Scale	3.04	0.84	.67	1–5	1.25–4.60

*Barclay Sick Role Process Inventory.

**Table 2:** Bivariate correlations of independent variables, control variables, and dependent variables.

Variable	1	2	3	4	5	6	7	8	9	10	11	12
(1) Goal	—											
(2) PsyTot	−518**	—										
(3) Som	−.277*	.478**	—									
(4) Dep	−386**	.754**	−.026	—								
(5) Anx	−439**	.865**	.453*	.389**	—							
(6) PreRig	.196	.036	.298*	−.189	.122	—						
(7) PreRes	.221	−.169	.020	−.308*	−.007	.145	—					
(8) PostRig	−.220	.310*	.315*	−.012	.443**	.431**	−.293*	—				
(9) PosRes	.224	−.367**	−.157	−.216	−.388**	.142	.380**	−.415**	—			
(10) LOF	−.042	.028	−.085	.076	.014	−.153	−.232	.010	−.318*	—		
(11) Time	−.010	.137	.166	.006	.102	.011	.088	.136	.015	−.044	—	
(12) Age	−.125	.070	−.003	.056	.075	−.292*	−.125	.139	−.368**	.222	−.120	—
(13) Gen	−.191	.428**	.430**	.026	.570**	.284*	.170	.350**	−.135	−.037	.049	.120

*P < .05.

**P < .01.

**Table 3:** Significant variables using stepwise regression analysis to predict psychosocial distress*.

Predictor	Beta	*t*	Significance level	Adjusted *R* ^2^
Goal attainment	−3.42	−3.87	.000	.256
Gender**	5.4	3.10	.003	.359
Postrehab response	−.651	−2.25	.029	.402

*F = 14.2, *R* ^2^ = .402.

**Lower score (1) = male; higher score (2) = female.

**Table 4:** Significant variables using stepwise regression analysis to predict goal attainment*.

Predictor	Beta	*t*	Significance level	Adjusted *R* ^2^
Anxiety	−.340	−2.73	.008	.179
Depression	−.254	−2.04	.046	.221

*F = 9.3.

## Appendix

### A. Barclay Sick Role Process Inventory

Instructions:

Please answer the following statements with (a)Strongly Disagree, (b)Disagree, (c)Agree, or (d)Strongly Agree.

Statements in Part I will focus on your beliefs and feelings you had at the beginning of your rehabilitation.
Statements in Part II will focus on your beliefs and feelings now.

Part I: Please Answer All of Questions in Part I Based on How You Felt at the Beginning of Your Rehabilitation

1. When I first started in this program, I had a right to be excused from all my daily responsibilities.
  - Strongly Disagree
  - Disagree
  - Agree
  - Strongly Agree
2. When I first started in this program, my family and friends should not have expected me to do as much for them as I did before my brain injury.
  - Strongly Disagree
  - Disagree
  - Agree
  - Strongly Agree
3. When I first started in this program, I expected that others care for and protect me.
  - Strongly Disagree
  - Disagree
  - Agree
  - Strongly Agree
4. When I first started in this program, I believed it was not my fault that I had a brain injury.
  - Strongly Disagree
  - Disagree
  - Agree
  - Strongly Agree
5. When I first started in this program, I deserved any disability benefits for which I was qualified (for example: time off from work or school or disability checks).
  - Strongly Disagree
  - Disagree
  - Agree
  - Strongly Agree
6. When I first started in this program, I thought my injury was a punishment for past sins.
  - Strongly Disagree
  - Disagree
  - Agree
  - Strongly Agree
7. When I first started in this program, my priority was to get back to work or school and my normal routine.
  - Strongly Disagree
  - Disagree
  - Agree
  - Strongly Agree
8. When I first started in this program, I was looking forward to getting back to work and my normal routine.
  - Strongly Disagree
  - Disagree
  - Agree
  - Strongly Agree
9. When I first started in this program, I wanted to get better than I was.
  - Strongly Disagree
  - Disagree
  - Agree
  - Strongly Agree
10. When I first started in this program, I believed it was important to get expert rehabilitation care.
  - Strongly Disagree
  - Disagree
  - Agree
  - Strongly Agree
11. When I first started in this program, it was important to me to regularly attend all rehabilitation and medical appointments.
  - Strongly Disagree
  - Disagree
  - Agree
  - Strongly Agree
12. When I first started in this program, it was important to me to follow all of my therapists' suggestions and apply those suggestions in my home life.
  - Strongly Disagree
  - Disagree
  - Agree
  - Strongly Agree

Part II: Please answer all of questions in Part II based on how you feel now

(1) Now, I have the right to be excused from all my daily responsibilities.
  - Strongly Disagree
  - Disagree
  - Agree
  - Strongly Agree
(2) Now, my family and friends should not expect me to do as much for them as I did before my brain injury.
  - Strongly Disagree
  - Disagree
  - Agree
  - Strongly Agree
(3) Now, I expect others to care for and protect me.
  - Strongly Disagree
  - Disagree
  - Agree
  - Strongly Agree
(4) Now, I believe it is not my fault that I have a brain injury.
  - Strongly Disagree
  - Disagree
  - Agree
  - Strongly Agree
(5) Now, I deserve any disability benefits for which I am qualified (for example: time off from work or school or disability checks).
  - Strongly Disagree
  - Disagree
  - Agree
  - Strongly Agree
(6) Now, I think my injury is a punishment for past sins.
  - Strongly Disagree
  - Disagree
  - Agree
  - Strongly Agree
(7) Now, my priority is to get back to work or school and my normal routine.
  - Strongly Disagree
  - Disagree
  - Agree
  - Strongly Agree
(8) Now, I am looking forward to getting back to work and my normal routine.
  - Strongly Disagree
  - Disagree
  - Agree
  - Strongly Agree
(9) Now, I want to get better than I am.
  - Strongly Disagree
  - Disagree
  - Agree
  - Strongly Agree
(10) Now, it is important that I get expert rehabilitation care.
  - Strongly Disagree
  - Disagree
  - Agree
  - Strongly Agree
(11) Now, it is important to me to regularly attend all rehabilitation and medical appointments.
  - Strongly Disagree
  - Disagree
  - Agree
  - Strongly Agree
(12) Now, it is important to me to follow all of my therapists' suggestions and apply those suggestions in my home life.
  - Strongly Disagree
  - Disagree
  - Agree
  - Strongly Agree

### B. Goal Attainment Scale

Primary therapist “Goal attainment scale” Name of client: Based on your prediction when the client first entered the rehabilitation program, for your specific discipline, please mark the outcome for this client

Level of Predicted Attainment

1. Most unfavorable outcome thought likely
  - Psychosocial —
  - Occupational —
  - Physical —
  - Cognitive —
2. Less than expected success
  - Psychosocial —
  - Occupational —
  - Physical —
  - Cognitive —
3. Expected level of success
  - Psychosocial —
  - Occupational —
  - Physical —
  - Cognitive —
4. More than expected success
  - Psychosocial —
  - Occupational —
  - Physical —
  - Cognitive —
5. Most favorable outcome thought
  - Psychosocial —
  - Occupational —
  - Physical —
  - Cognitive —
